# Health in Our Homes

**Published:** 1889-02-16

**Authors:** Herman Laurence


### Health in Our Homes.

By Herman Laurence, M.R.C.P., and L.R.C.S.E., etc.

During the two years I have spent on this side of the world I have devoted a good deal of attention to the sanitary arrangements of hospitals, and have also been called upon to visit the poor; so that I have had ample opportunity, sought and unsought, to study sanitation at its best and worst. These studies are the foundation of the opinions I hold, and the laws I desire to explain concerning Health in our Homes.

The greater part of my experience has occurred in Scotland, where the flat system, recently introduced in London, has been in full swing for countless generations; only in Edinburgh they do not speak of living in a flat, they say they live "up a common stair." Anyone who has gone up these common stairs, either in London or in Scotland, must be well acquainted with the close and disagreeable atmosphere that is met there, and how very rare it is to find any improvement in these respects on entering the dwellings of the poor. The "stuffiness" is almost unendurable to any one who is used to even a moderate amount of air. It is needless to say how injurious this is to all who live in these places, and it is often the result of the ignorance or carelessness of the elder and more responsible members of the family. Sometimes it is the builder's fault, but not often. Let us give even the "jerry builder" his due, and admit that he oftener affords unnecessary facilities for the entrance of air by means of ill-fitting windows and doors, than does anything to prevent some sort of ventilation. Unfortunately the air that comes in from the common stair is by no means of the best quality, and other means of ventilation must be sought. Where it is practicable it is well to put in a Tobin's tube in the wall opening on the outer air at the lower, and in the room at the upper end. In some houses you will find that this has been done in the building, in which case it is wise to explain to the inhabitants that the grating in the wall should not be choked up with a duster or have brown paper pasted over it. I have seen both methods of rendering the tube useless practised.

Where no such means of ventilating an apartment has been provided, a good deal can always be done towards purifying the atmosphere by a good fire, which draws the foul air up the chimney, and when the impure air is thus drawn away other air will force its way in by every nook and cranny, because, as we were all taught at school, nature in many instances seems to abhor a vacuum. It should be more generally known that impure air, being heated and containing the waste products of respirations, rises to the ceiling, and therefore escapes more easily if the window is opened from the top. I know a boarding-school where small blocks of wood are fixed at the upper part of the window in such a fashion as to prevent the windows closing for the space of one inch—just sufficient to provide a continuous free exit for impure air. Pure air, being colder than the other, enters by preference at the lower part of the room, and if its entrance is provided for simply opening the window at the bottom, it is likely to be felt, in small rooms at least, in the unpleasant form of draught. This, however, can be remedied by fixing a slanting board in the open window to guide the air upwards over the heads of those in the room. It then circulates imperceptibly, without being felt as a draught.

The more air there is provided for each person the less frequent is the need of changing it, and therefore the more gentle and imperceptible the current of fresh air may be. In hospitals there is allowed from two to three thousand cubic feet of air, changed once an hour, to each bed; but in most lodging-houses three to four hundred feet is the utmost allowed, and in the poorer sort very much less. Where 500 feet are allowed for each person, the air should be changed three or four times an hour, and when the ratio is lower, and many people are sleeping in one flat, it would require nothing less than a decided draught to keep the air at all sweet. It is popularly taught that carbonic acid gas is the poisonous ingredient in expired air—that is, air that has been inhaled into the lungs, and rendered impure by being breathed out charged with waste products from the body. This is hardly accurrate. Carbonic acid is present, but, at least in Sir Henry Thompson's opinion, it acts only as an index to the amount of organic matter in the air, which is the really poisonous element. Air composed of a large proportion of carbonic acid gas might be inhaled with impunity if it were not for the organic substances that invariably adulterate it.

Air, being the first essential of life, is also the first essential to health, but very near it comes cleanliness, both in regard to the person and the house. Attached as I am to Scotland, I nevertheless must admit that it is a country where personal cleanliness is shamefully neglected among the lower classes; and even if things are a degree better in the South, there is, I fear, much room for improvement. The function of the skin is not generally understood by the public, and I should like to impress on every one the necessity for keeping that function healthy. Those doctors who, like myself, devote special attention to diseases of the skin, know what serious disorders are apt to result from its neglect. A healthy skin is a sure criterion of good health, and the rosy cheek of the milkmaid as surely tells of her good physical condition as the pale cheeks of our townsfolk speak of the weakness brought on by bad air and insufficient or unsuitable food.

One of the functions of the skin is to regulate the temperature of the body. About 80 per cent. of the heat of the body is lost by the skin, so it might be thought that people who indulge in a good thick layer of dirt should have an advantage in cold weather. This is not so, however, for one of the ways in which the skin loses heat is by conduction, and dirt is an admirable conductor. Moreover, the presence of dirt prevents the blood-vessels of the skin acting properly. In a healthy condition they contract under cold, keeping in the warmth, and dilate with heat, letting it out, and so keep up an equable temperature in the body in spite of all variations of outside temperature. Now, anything that interferes with the function of the skin prevents the action of this compensating power. If you were to varnish the body all over death would quickly result from blood-poisoning, accompanied by the lowering of the temperature. It is by means of this continuous evaporation from the skin, which we call perspiration, that men whose occupations call them to live in hot climates, or elsewhere, under high temperatures, are able to stand the heat. This was conclusively proved by an experiment made by Sir Charles Blaydon, who remained for eight minutes in an atmosphere of 260 deg. Fahrenheit, with a leg of mutton cooking beside him. As the boiling point of water is 212 deg. this seems absolutely incredible, but the experiment really took place, and without injurious results.

The skin being thus such an important organ, it is eminently desirable that its beneficent work should not be impeded, and the primary object of a bath is to clear the way from all impediment. For cleansing purposes a hot bath is of course the best; but as this leaves the blood-vessels of the skin—the capillaries, as they are called, from their hair-like fineness — in a dilated condition, in which any draught of cold air is likely to be injurious, therefore, they should not be indulged in oftener than is necessary for cleanliness—say, once or twice a week—and should be taken at bedtime, when there is little chance of exposure to cold. A cold bath, on the other hand, strengthens and stimulates the skin, keeps the capillaries in good working order, sends the blood coursing through the body at an accelerated rate, and therefore forms a good preparation for each day's work. Of course there are people who cannot stand a cold bath, whom it leaves blue, cold, and miserable, instead of warmed and exhilarated. For these a bath "with the chill off" —just a little colder than tepid—is suitable; but no mortal in an average state of health should evade the duty of washing thoroughly once a day. Of course the poor protest that they have no bath rooms at home, and cannot afford to pay the moderate prices asked at our public baths; but a man who wants to be clean will be clean in spite of difficulties, and a curtain fastened across a corner of the room, with a washing tub behind it, will form an efficacious substitute for the ordinary bath.

I should like to say a word about bed-clothes. Heavy bed-clothes are unhealthy, and conduce to evil dreams; yet in really cold weather it is almost impossible to obtain sufficient heat without too great a weight of blankets. In such circumstances an eider-down quilt is of course an admirable substitute, but we cannot all afford eider-downs. Therefore, as the next best thing I would suggest an Indian rezui, The materials for this are four yards of cotton-wadding, and some sheeting, printed cotton, or cretonne by way of covering. The wadding is about three-quarters of a yard wide, and is double. Open it, and fold it the other way, so that instead of four yards of material three-quarters of a yard wide, you have two yards of a yard and a-half wide; enclose this in the covering you have chosen, and you have a light and warm quilt at a cost of little more than a shilling. It is as light as a down quilt, cheaper, and superior to it in that it allows of ventilation. All bed-coverings should permit evaporation, or they will be unhealthy, and it is not wise to get into the habit of being too warm in bed. Too warm sleep does less good, is less refreshing, than that enjoyed in a moderate temperature.

With regard to food, it is essential that meals should be regular. The time for them must vary with different people to suit various occupations; but whatever hours are chosen should be adhered to with regularity. McCall Anderson says: There should be five hours between each meal, in order that one may be digested before another is begun. With regard to the kind of food, it must depend greatly on the means of the eater, and it must be remembered that it is possible to get cheap things that shall be quite as good food-value as all the "luxuries of the season," or better. In Australia, it must be admitted that the working man can get more food for his money than he can here. In a Melbourne restaurant the following bill of fare was advertised for sixpence: Meat (and meat being cheap there is helped with great liberality), with potatoes and another vegetable, tart, cheese, a glass of beer, and a game of billiards.

Tea as an article of food is much abused, especially by women. It has a certain stimulating power, but its effect is to dull the appetite, and therefore prevent the consumption of more nourishing food. This causes emaciation of the whole system, and in many cases of anæmia it would not be bad treatment to forbid the patient to partake of tea. She would then recover her natural appetite, and the bloodlessness and emaciation would disappear. With regard to starchy foods of all sorts, Dr. McCall Anderson impresses the necessity of chewing them well in the mouth. This enables the saliva to act on them and helps digestion. The stomach always has enough to do without meddling with slack. The poor gastric juice is overworked in various way; I may mention one. Parents often give their children milk to drink with meat, under the impression that it is a harmless drink suitable for youth. Milk is a food, and the casein it contains becomes a solid on entering the stomach. Its alteration into a fluid, suitable for absorption into the blood, occupies all the energies of the gastric juice, and the digestion of the meat is interfered with. The feeding of children is always a matter to be dealt with carefully, and it cannot safely be trusted, as many people think, to parental instinct. A story is told of a gentleman, travelling in a railway carriage, remonstrating with a female fellow-passenger who was feeding her baby with a piece of raw herring. "Excuse me, sir," replied the mother with dignity, "I think I ought to know better than you; I've buried seven!" Doubtless before long she would be able to boast of having buried eight.

I will say a word in conclusion as to furniture. Carpets, though necessary in a climate like ours, at least in winter, are great harbourers of dust, and, therefore, may become injurious to health. It is needless to state that they should be well and frequently swept, but the process of sweeping may do harm by raising the dust and scattering it about the room. Anyone entering the room while this dust is floating in the air will be liable to inhale it, though if it has had time to settle, the movement of persons about the room will not again raise it. It is desirable, therefore, that a carpet should be swept a considerable time before the room is occupied, or if this is impracticable, there is nothing better than the time-honoured plan of scattering damp tea leaves on the carpet before sweeping it. The dust then adheres to the surface of the leaves and does not rise at all. The present fashion of having a carpet only in the centre of the room, with two or three feet of painted or varnished wood between it and the wall is decidedly to be commended on hygienic grounds.

In the space at my command it is not possible for me to do more than give a few hints. Much has been omitted, much I must assume your own common-sense has taught you; but I trust I have said enough to suggest to you one or two ways to attain Health in our Homes.

